# Supplementary material for: Cement substitution with secondary materials can reduce annual global CO2 emissions by up to 1.3 gigatons
Source: Nat Commun. 2022 Sep 30;13:5758. doi: 10.1038/s41467-022-33289-7 (PMC9525259; doi:10.1038/s41467-022-33289-7)
Supplement: Supplementary file 1 — Supplementary Information S1 [file 41467_2022_33289_MOESM1_ESM.pdf]

Supplementary information for:

Cement substitution with secondary materials can reduce annual global CO_2 emissions by up to 1.3 gigatons

Izhar Hussain Shah [1], Sabbie A. Miller [2], Daqian Jiang [3], Rupert J. Myers [1,*]

[1] Department of Civil and Environmental Engineering, Imperial College London, Skempton Building, London, SW7 2AZ, UK

[2] Department of Civil and Environmental Engineering, University of California, Davis 2001 Ghausi Hall, One Shields Ave, Davis, CA, 95616

[3] Department of Civil, Construction, and Environmental Engineering, University of Alabama, Tuscaloosa, AL, 35401, USA

* Corresponding author email: r.myers@imperial.ac.uk.

Table of contents

S1. Supplementary methods S3
S1.1. Availabilities of secondary cementitious materials S3
S1.1.1. Coal fly ash S3
S1.1.2. Flue gas desulphurisation by-products S3
S1.1.3. Granulated blast furnace slag S4
S1.1.4. Silica fume S5
S1.1.5. Bauxite residue S6
S1.1.6. Agricultural by-product ashes S6
S1.1.7. Forestry by-product ashes S7
S1.1.8. End-of-life binder S8
S1.2. Life cycle assessment S9
S1.2.1. Product system S9
S2. Supplementary discussion S16
S2.1. Life cycle assessment contribution analysis S16
S2.2. Life cycle assessment sensitivity analysis S18
S3. Supplementary references S19

S1. Supplementary methods

S1.1. Availabilities of secondary cementitious materials

S1.1.1. Coal fly ash

Coal-fired electricity generation produces several by-products that can be used as substitutes for Portland cement (PC) clinker. Coal fly ash (often referred to as 'fly ash' or 'pulverised fuel ash') is the most important. Coal fly ash has been utilised as a cementitious material (CM) for almost one century, which was first reported in the 1930s[1], and is currently one of its main uses alongside land reclamation[2,3]. The chemical and physical properties of coal fly ash depend on the coal type and processing method. Two main classes of fly ash exist ('siliceous', i.e., deficient in calcium, and 'calcareous', i.e., rich in calcium[4]). Both classes are reactive ('pozzolanic') and thus can be used as CMs.

Generation of coal fly ash (~0.35 Gt per year, Supplementary Fig. S1) is significantly less than current PC production (~4 Gt in 2018[5]). Therefore, our results demonstrate that coal fly ash is suitable for common use averaging at a minor to moderate CM to cement ratios <~0.2. Due to the success of coal fly ash as a CM, its utilisation in cementitious materials is likely to continue while coal-fired electricity generation remains a significant component of energy supply. Coal-fired electricity generation is expected to remain stable or increase in the short term[6], indicating similar or slightly greater generation rates of coal fly ash in the 2020s.

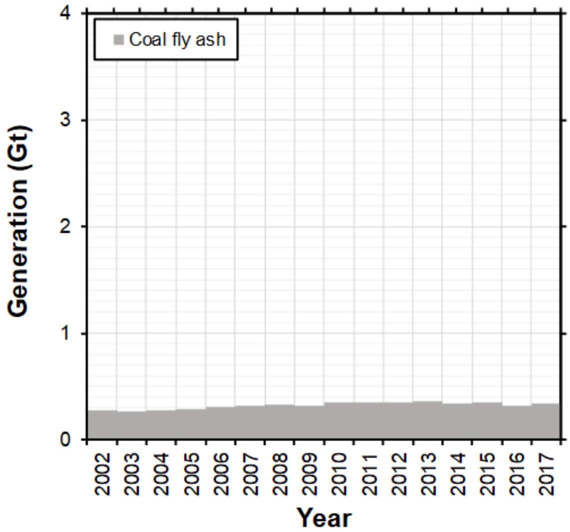

Supplementary Fig. S1. Generation of fly ash from coal-fired power plants, globally (2002–2018), plotted in the range relative to current PC production (~4 Gt in 2018).

S1.1.2. Flue gas desulphurisation by-products

Natural and flue gas desulphurisation (FGD) gypsum perform similarly in cement. The latter is derived from burning of fossil fuels. The S (elemental sulfur) contents of fossil fuels are generally minor but non-zero, with typical S concentrations of <11 mass% in coal[7], <5 mass% in crude oil[8], and trace levels in natural gas[9]. Coal and crude oil are thus the two most important fossil fuels with respect to overall S content. Currently, ~12 Gt of coal (~8 Gt) and crude oil (~4 Gt) are extracted annually (in 2017[10]), which each contribute ~30% to total primary energy supply (in 2015)[11]. When burned, these fuels release gaseous S as SO_x. Such

emissions have been increasingly targeted in environmental policy since the 1960s, limiting the amounts of SO_x emitted per unit of energy produced and S content in products such as diesel fuel[8].

Presently, SO_x gases are scrubbed with wet/limestone slurries to produce gypsum in coal FGD systems[12], whereas elemental S is produced from gaseous H_2S in crude oil refining FGD systems (the Claus process)[8]. Elemental S is mostly used to produce sulfuric acid (H_2SO_4), whereas FGD gypsum is mainly used in plaster/wallboard production (62% in 2013), and to a lesser extent cement (9% in 2013) in other applications[13]. FGD gypsum is preferred over natural gypsum and anhydrite in plaster/wallboard manufacturing, with two key reasons being: economic; and since plaster/wallboard raw materials are first processed to the basanite composition (and then rehydrated to set into a board), preferably by dehydrating gypsum, making anhydrite less desirable for this end use. Increasing amounts of FGD gypsum have been produced in recent years, with ~0.3 Gt of FGD gypsum produced in 2017 from coal-fired power generation (Supplementary Fig. S2).

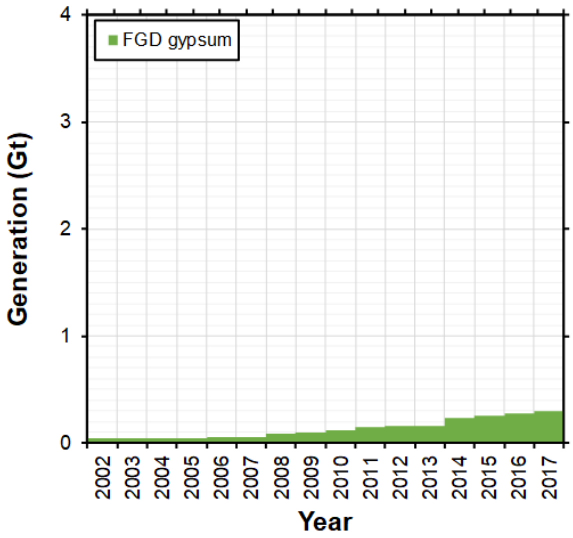

Supplementary Fig. S2. Generation of FGD gypsum from coal-fired power plants, globally (2002–2018), plotted in the range relative to current PC production (~4 Gt in 2018).

FGD gypsum is currently only partially utilised (50-60% is discarded in the United States[14]). This situation of high FGD availability is likely to continue in the short term (i.e., years) due to the high fraction (~30% in 2015) of global energy supply from coal[11], but would decline if coal is substituted for alternative forms of energy supply, e.g., renewables. A substantial decrease in the use of coal-fired electricity production (on the order of a halving of its current use due to the low utilisation rate of FGD gypsum) would drive an increase in mining of natural gypsum/anhydrite. Significantly increasing utilization of calcium sulfate in cement may also drive an increase in mining of natural gypsum/anhydrite. Therefore, the availability of FGD gypsum (similar to natural calcium sulfate resources) are sufficient to meet demand for the foreseeable future.

S1.1.3. Granulated blast furnace slag

Slags generated as by-products during metal smelting can be used as CMs, of which the most important is blast furnace slag. Blast furnace slag is a by-product of pig iron production. It is either (slowly) air-cooled to form dense and hard aggregate that is suitable for use as

aggregate, e.g., in road-base or asphalt concrete[15], or (rapidly) quenched to form granulated or pelletised material that can be further ground for use as a CM[15]. The latter material is called ground granulated blast furnace slag (GBFS) and has been utilised in cements since the 1860s[16].

Our results, which show that ~0.3 Gt blast furnace slag was produced in 2018 (Supplementary Fig. S3), are an order of magnitude smaller than PC production rates (~4 Gt in 2018[5]). Ground GBFS is thus suitable for common use averaging at a minor secondary CM to cement mass ratios of <~0.1. In the future it is expected that relatively more steel will be produced from steel scrap rather than primary iron ore[17], reducing potential GBFS generation, and maintaining its status as a commonly used minor CM at the global scale.

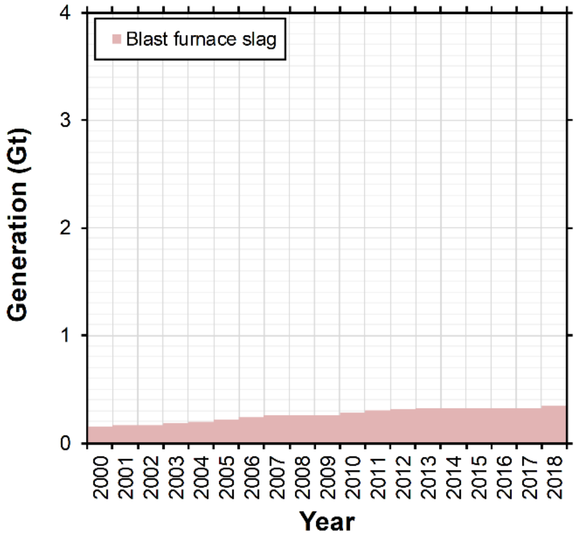

Supplementary Fig. S3. Generation of blast furnace slag, globally (2000–2018), plotted in the range relative to current PC production (~4 Gt in 2018).

S1.1.4. Silica fume

Silica fume, which is produced during silicon and ferrosilicon alloy manufacturing, has been used as a CM since the 1970s due to its pozzolanic properties[16]. It comprises spherical particles (typical individual diameters of 0.01–0.1 μm) of mostly amorphous SiO_2 (usually >85 mass% SiO_2), which agglomerate to form larger particles with typical diameters of up to hundreds of μm [18,19]. Silicon and ferrosilicon alloys are typically produced from the following raw materials: high purity SiO_2 sources such as sand and gravel; C sources such as coke or coal; and Fe sources such as iron ore or scrap. In silicon and ferrosilicon alloy production these raw materials are fed into an electric arc furnace, from which a reduced Si product is tapped into a ladle and refined to remove impurities such as Al and Ca (in a slag), and then cast[20]. Silica fume is produced in each of these three manufacturing steps. It is captured from the electric arc furnace and ladle during tapping and refining, and both sources of silica fume have similar chemical and physical properties[21]. Silica fume generated when the ladle is removed from the tapping area to the casting area is not captured[20], but potentially can be used as a CM.

Production of the reduced Si product in an electric arc furnace generally follows the idealised reaction shown in eq.(1):

$$SiO_2(s) + 2C(s) \leftrightharpoons Si(l) + 2CO(g) \quad (1)$$

We calculate a total potential generation of silica fume of ~0.003 Gt/year over the period 2008–2018 (which is the same order of magnitude as other estimates[22]), which is three orders of magnitude lower than current rates of PC production (~4 Gt in 2018[5]). Therefore, silica fume is and will remain a minor CM for the foreseeable future.

S1.1.5. Bauxite residue

The Bayer process is used for industrial production of alumina (i.e., aluminium oxide, Al_2O_3), which involves the conversion of bauxite ore to alumina and bauxite residue. This process dissolves the aluminium-containing minerals in bauxite ore in a highly alkaline aqueous NaOH solution at elevated temperature (100-350 °C) and pressure[23]. The product, a pregnant aluminium-rich liquor, is separated from bauxite residue, which is the by-product solid material. Additional unit operations are usually employed to recover aqueous NaOH solution, which is returned to the process. Bauxite residue is usually alkaline, Fe and Al rich, contains significant water content, and usually requires treatment, e.g., calcination at 600-800 °C, to be cementitious[24].

In 2018, 131 Mt alumina was produced globally, yielding ~155 Mt bauxite residue using a bauxite residue to alumina mass ratio of 1.19 (the world average value). This amount of bauxite residue is an order of magnitude lower than PC production rates (~4 Gt in 2018[5]), thus bauxite residue is suitable at minor global average secondary CM to cement mass ratios of <~0.1.

S1.1.6. Agricultural by-product ashes

Agricultural crops, by-products, and residues, i.e., the parts of crop plants that are non-edible for humans, are among the most produced materials[10]. In 2018, the most produced crops included sugar cane (1.9 Gt), maize (1.1 Gt), paddy rice (0.78 Gt), and wheat (0.74 Gt)[25]. Crop by-products may be generated in the field (e.g., straw) or downstream processing (e.g., rice husks). Crop by-products have various end uses, including fodder, soil enhancement, and energy recovery[26]. The latter end use is especially relevant in the CM context, since energy recovery can produce desirable ash type by-products, derived mainly from unburned/mineral components. In general, these ashes are pozzolanic and rich in SiO_2. Rice husk ash is a particularly notable example that has been studied for decades[27] and can contribute to desirable environmental sustainability goals when used as a CM[28].

Our results show that a maximum of ~0.9 Gt/year of crop by-product ashes may be currently produced worldwide (ca. 2018, Supplementary Fig. S4), which is approximately of the same order of magnitude but smaller than PC production (~4 Gt in 2018[5]). The agricultural by-products with the highest potential ash generation are rice straw (~0.26 Gt ash in 2018), maize stalk (~0.24 Gt ash in 2018), and wheat straw (~0.14 Gt ash in 2018). Therefore, agricultural by-product ashes can currently be used as CMs at minor to moderate substitution levels at the global level, i.e., they are borderline high availability CMs. However, prevailing competition for crop by-products as feedstocks for other end uses indicates that these materials may only achieve minor PC substitution levels if commonly used.

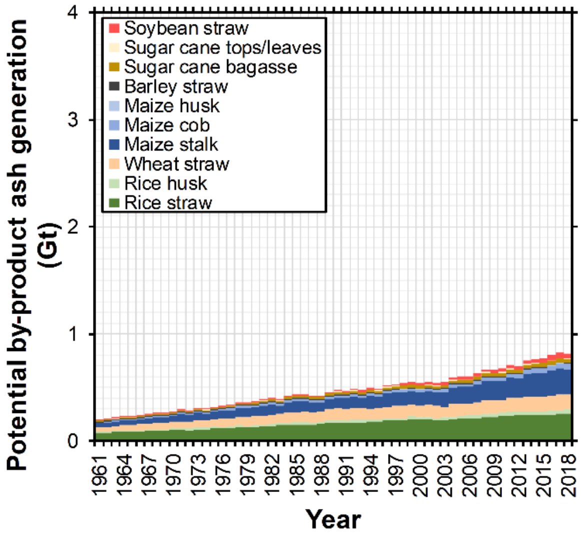

Supplementary Fig. S4. Potential generation of ash from several of the most highly produced crop by-products (legend), globally (1961–2018), plotted in the range relative to current PC production (~4 Gt in 2018). The data shown here were calculated using FAO statistics[25], reported main product-to-by-product ratios and ash contents[29], and assuming all crop by-products are recovered and treated by energy recovery.

S1.1.7. Forestry by-product ashes

Wood is a common material, albeit produced in lower quantities (~2.3 Gt in 2017) than agricultural crops and other materials[10], that is mostly used in durable timber products and fuel. We denote these products, classified under 'roundwood' [25], as main forestry products. By-products from logging processes, 'forest residue', include branches, tops, unmarketable logs, etc., and are usually collected for energy recovery, which ultimately generates ash by-products. Ashes generated from burning woody material are typically rich in SiO_2 and/or CaO[30], indicating their potential to be used as CMs.

Our results show potential ash generation values of ~0.1 Gt in 2018 (Supplementary Fig. S5). This value is an order of magnitude lower than cement production (~4 Gt PC in 2018[5]), demonstrating the potential to use forestry by-product ashes as CMs at low substitution extents only, at the global scale. Significantly lower quantities of by-products are generated downstream of forestry operations, e.g., during saw-milling, although this woody material may also be used for energy recovery and leading to generation of ash by-products (Supplementary Fig. S5).

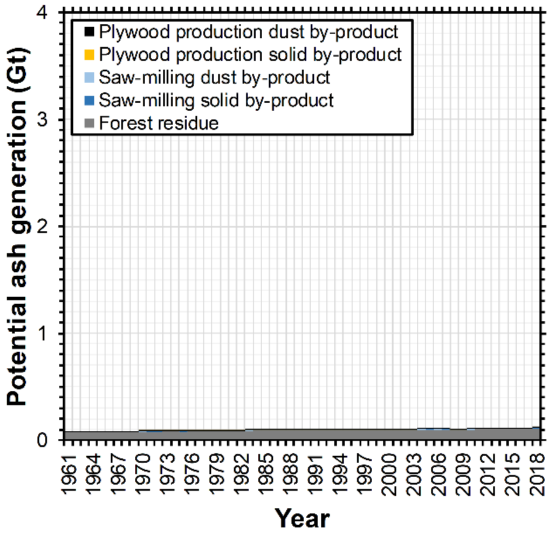

Supplementary Fig. S5. Potential generation of ash from several of the most highly produced forestry by-products (legend), globally (1961–2018), plotted in the range relative to current PC production (~4 Gt in 2018). The data shown here were calculated using FAO statistics[25], reported main product-to-by-product ratios and ash contents[29], and assuming all forestry by-products are recovered and treated by energy recovery.

S1.1.8. End-of-life binder

The constituents of construction and demolition waste (mortar and concrete) include secondary aggregate (fine and coarse, ~65-81 mass%) and end-of-life binder (~19-35 mass%)[31]. Although secondary aggregate and end-of-life binder are not typically separated at present, i.e., most end-of-life concrete is used as unseparated secondary aggregate in loose applications such as road sub-base[32,33], the latter (end-of-life binder) can be used as a CM. It is a heterogeneous material, comprising (hydrated and) carbonated binder (typically ~13-24 mass%), of which typically ~4-8 mass% is hydrated (and uncarbonated) binder and ~2-4 mass% is unhydrated cement[31]. The former, carbonated material contains a significant amount of $CaCO_3$ and thus can be expected to behave somewhat similarly to limestone as a CM, whereas the latter fraction of end-of-life binder is unhydrated cement and so would behave similarly to the cement used in the initial mix.

Most CM entering use at a given point in time will only reach end-of-life decades later, e.g., a concrete product used in new construction in 2020 will most likely reach end-of-life after 2050: typical lifetimes of cementitious material products, i.e., buildings (residential and non-residential) and infrastructure (e.g., roads), are estimated between 30 and 70 years[34]. Therefore, the availability of CMs in construction and demolition waste during the next few decades will depend mainly on the historical use of mortar and concrete.

Our estimates of the amounts of binder (i.e., cement + water; in uncarbonated form) removed from use at end-of-life annually show increasing trends, reaching a maximum of 1.3 Gt during the period 2002-2018 (Supplementary Fig. S6). Therefore, end-of-life binder is currently suitable for use as a CM at moderate substitution rates. In the future, we expect more end-of-life binder to be generated relative to cement production rates due to the nature of stock-flow dynamics of cementitious materials (an increasing amount will reach end-of-life in the future due to the historical accumulation of the in-use stock), which indicates that

this material will eventually have the potential to be used as a CM at higher substitution levels.

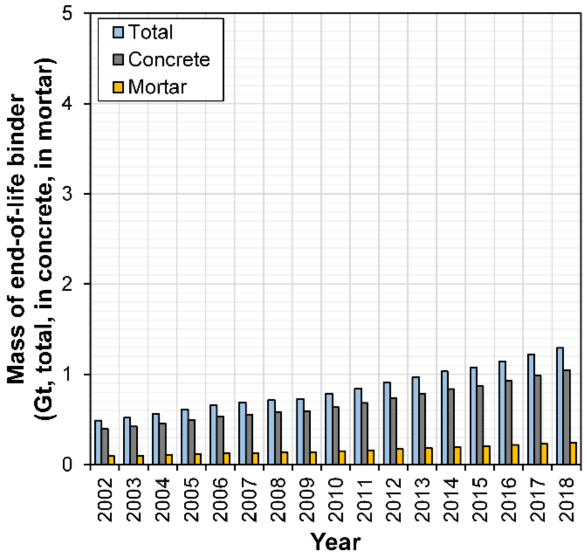

Supplementary Fig. S6. Potential generation of end-of-life binder (i.e., cement + water) removals of cement from the in-use stock (in concrete, in mortar, and total), globally (2002–2018), plotted in the range relative to current PC production (~4 Gt in 2018). The data shown here were produced using a dynamic material flow analysis model, concrete and mortar binder intensities, and typical cement end use percentages, as described in the text.

In this work, the magnitude of end-of-life CMs produced annually is based on modelling inputs from Olsson *et al.* [35] and Cao *et al.* [36]. Namely, from these studies, per capita cement saturation values (i.e., the highest level of cement demanded per person in each region) and cement in-use stock lifetimes were taken. Using global population statistics and projections [37,38], the amount of cement produced annually (inflows of cement into the in-use stock, *i*), the differences in flow (a netflow of cement into the in-use stock, *n*), and the cement removed annually (outflows of cement from the in-use stock, *o*) were estimated. The inflows and netflows of cement into the in-use stock were based on the application of a Gompertz combined model, as was stipulated in Olsson *et al.* [35] and Cao *et al.* [36]. We note that this modelling method smooths out year-on-year variations that are measured in annual cement production reports from organizations like the United States Geological Survey[5]. The cement outflow from the in-use stock was determined as the net difference between these flows, that is $o = i - n$. This calculation method for the outflow of cement from the in-use stock was performed for each of the 10 global regions used by Cao *et al.* [36], i.e., North America, Latin America & Caribbean, Europe, Commonwealth of Independent States, Africa, Middle East, India, China, Developed Asia & Oceania, Developing Asia (referred to within this work as Other Asia), in order to maintain necessary consistency for per capita cement saturation values and cement in-use stock lifetimes. We calculated amounts of cement binder removed annually from the outflows of cement from the in-use stock using our data for cement and binder intensities, and typical percentages of cement used in mortar and concrete.

S1.2. Life cycle assessment

S1.2.1. Product system

The generic product system used in the LCA models is shown in Supplementary Fig. S7. Region-specific LCA models were developed for Brazil, Canada, China, India, Japan, South Korea, Turkey, United Kingdom, and the United States. These nine countries, representing ~70% of global cement production in 2018, are considered to represent world cement production, and consequently the GHG emissions reductions based on clinker substitution, when scaled globally. Clinker production is the most CO_2 emissions intensive process in cement manufacturing, and ecoinvent[39] (version 3.6, cut-off system model) includes country-specific system processes for clinker production i.e. containing all associated upstream processes/impacts including the construction of production facilities. These available clinker production processes in ecoinvent[39] (version 3.6, cut-off system model) are based on country-specific data enabling good regional representation of our LCA results. For other processes, when country-specific inventory data were unavailable, the most appropriate datasets providers were used (e.g., Europe and global, respectively).

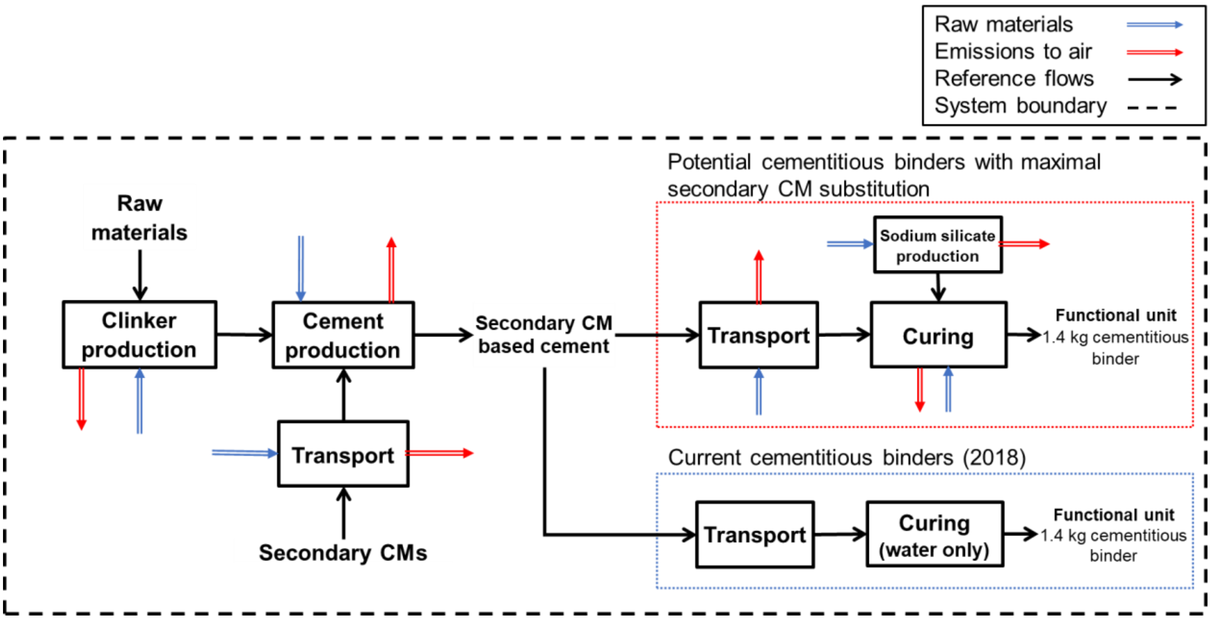

Supplementary Fig. S7. Scope of the LCA analysis and system boundary. The product system is based on the existing business-as-usual scenario (excluding the red dotted box) where current fractions of clinker, gypsum, and secondary CMs (mainly coal fly ash and granulated blast furnace slag, including a 150 km of secondary CM transport) are used to produce 1.4 kg of cementitious binder. The red dotted box shows the production of potential cementitious binders with maximal secondary CM substitution.

Our inventory analysis models are based on two types of cement binders:

i. An existing business-as-usual scenario where current fractions of clinker, gypsum, and secondary CMs (mainly coal fly ash and granulated blast furnace slag based on the Getting the Numbers Right database[40]) are used in the cement mix, and including 150 km of secondary CM transport from the point of generation to the cement plant and another 150 km from cement plant to the concrete batching plant (freight transport assumed on a Euro 5 type truck with a nominal payload capacity ≥32 tons). The concrete batching plant is where the addition of 0.4 kg of water with cement takes place, resulting in the production of the functional unit which is 1.4 kg cementitious binder (i.e., clinker, gypsum, secondary CMs, and water, as shown in Supplementary Table S1). No addition of sodium silicate activator takes place in this scenario.

ii. A high substitution secondary CMs scenario where all the locally available secondary CMs are assumed to substitute clinker and/or cement including 150 km transport from the point of generation to the cement plant (freight transport assumed on a Euro 5 type truck with a nominal payload capacity ≥32 tons), and an additional hypothetical activation step. The hypothetical activation step is included for all potential cementitious binders with maximal secondary CM substitution to ensure good cement reactivity. For this activation step, the functional unit (i.e., 1.4 kg cementitious binder) is produced at the concrete batching plant. The binder contains 0.926 kg of clinker, gypsum, and secondary CMs, 0.074 kg of (anhydrous) solid sodium silicate (i.e, the activator), and 0.4 kg of water, which is based on the mix design in [41]. The inventory data for solid sodium silicate was based on the ecoinvent system process "market for sodium silicate, solid". Since the activation step is assumed to take place at a concrete batching plant, 150 km of road freight transport is included from the cement plant to the concrete batching plant (where high secondary CM-containing cementitious binders may be activated and applied in end-use applications).

Supplementary Table S1 shows the shares of clinker, gypsum, secondary CMs, solid sodium silicate activator, and water considered in (a) current 2018 cementitious binder production, and (b) potential cementitious binder production with maximal substitution of clinker with locally generated secondary CMs. These maximally substituted cementitious binder mixes are modelled in our LCA study. Supplementary Table S2 shows the disaggregation of the secondary CMs (in mass%) in each country for the year 2018.

Supplementary Table S1

Fractions of clinker, gypsum, secondary cementitious materials (CMs), sodium silicate (anhydrous), and (mixing) water in the functional unit (1.4 kg cementitious binder) based on **(a)** cement production in 2018 and **(b)** potential cementitious binder production with maximal secondary CMs, by country. Cement production by country is also shown.

	**(a)**					
	Cement production, Gt	Clinker, kg	Gypsum, kg	Secondary CMs, kg	Activator, kg	Water, kg
Brazil	0.054	0.694	0.037	0.269	0	0.40
Canada	0.014	0.777	0.041	0.183	0	0.40
China	2.208	0.813	0.043	0.144	0	0.40
India	0.298	0.698	0.037	0.266	0	0.40
Japan	0.055	0.813	0.043	0.144	0	0.40
South Korea	0.058	0.813	0.043	0.144	0	0.40
Turkey	0.073	0.752	0.040	0.209	0	0.40
United Kingdom	0.009	0.774	0.041	0.186	0	0.40
United States	0.088	0.872	0.046	0.082	0	0.40
	**(b)**					
	Secondary CMs, Gt	Clinker, kg	Gypsum, kg	Secondary CMs, kg	Activator, kg	Water, kg
Brazil	1.763	0	0	0.926	0.074	0.40
Canada	3.692	0	0	0.926	0.074	0.40
China	0.521	0.444	0.023	0.459	0.074	0.40
India	0.979	0.019	0.001	0.906	0.074	0.40

Japan	2.459	0	0	0.926	0.074	0.40
South Korea	2.176	0	0	0.926	0.074	0.40
Turkey	0.705	0.273	0.015	0.639	0.074	0.40
United Kingdom	1.367	0	0	0.926	0.074	0.40
United States	4.871	0	0	0.926	0.074	0.40

Note: The amount of gypsum is based on a clinker to gypsum ratio of 95:5.

Supplementary Table S2

Disaggregation of the masses of secondary cementitious materials (CMs) that could have potentially been generated in year 2018 in a selection of countries, %

	Brazil	Canada	China	India	Japan	South Korea	Turkey	United Kingdom	United States
Flue gas desulphurization gypsum	0.7%	2.8%	13.0%	12.7%	6.7%	6.2%	6.6%	4.4%	8.9%
Coal bottom ash	0.2%	0.8%	3.6%	3.6%	1.9%	1.7%	1.9%	1.2%	2.5%
Coal fly ash	0.7%	3.1%	14.3%	14.0%	7.4%	6.8%	7.3%	4.9%	9.9%
Granulated blast furnace slag	8.3%	3.7%	18.4%	6.8%	15.6%	10.4%	5.7%	12.2%	1.5%
Bauxite residue	13.1%	3.7%	5.3%	2.2%	0.1%	0.0%	0.4%	0.0%	1.2%
Maize stalk ash	18.3%	5.8%	4.7%	2.0%	0.0%	0.0%	2.3%	0.0%	19.2%
Maize cob ash	3.4%	1.1%	0.9%	0.4%	0.0%	0.0%	0.4%	0.0%	3.6%
Rice straw ash	4.1%	0.0%	6.1%	19.4%	2.3%	1.4%	0.6%	0.0%	0.8%
Rice husk ash	0.6%	0.0%	0.9%	2.8%	0.3%	0.2%	0.1%	0.0%	0.1%
Soybean straw ash	16.1%	0.0%	0.2%	0.6%	0.0%	0.0%	0.0%	0.0%	3.7%
Sugar cane bagasse ash	16.0%	0.0%	0.2%	2.6%	0.0%	0.0%	0.0%	0.0%	0.0%
Wheat straw ash	1.1%	11.8%	2.1%	6.4%	0.1%	0.0%	7.3%	20.1%	2.2%
Forest residue ash	8.2%	8.4%	0.8%	3.3%	0.6%	0.1%	1.5%	2.5%	2.8%
End of life binder (mortar)	1.3%	10.8%	5.4%	4.2%	12.3%	13.8%	12.2%	9.7%	8.1%
End of life binder (concrete)	5.7%	46.0%	23.0%	18.0%	52.3%	59.1%	52.3%	41.2%	34.5%
Other secondary CMs	2.2%	1.9%	1.0%	1.0%	0.4%	0.3%	1.4%	3.8%	0.9%

Note: The sum of all potential secondary CM generation per country adds up to 100%

To assess the suitability of national average cementitious binder mixes for alkali-activation, we multiplied the mass percentages of each secondary CM in these mixes (excluding ‘other secondary CMs’) with reported oxide compositions (after scaling up the compositions to 100 mass%, Supplementary Table S3), to determine their average compositions. The results of this calculation (Supplementary Table S4) shows that the average compositions of the national average cementitious binder mixes are close to or lie within the ranges of the main components (SiO_2, CaO, Al_2O_3, MgO, Fe_2O_3) in typical oxide compositions of metakaolin, coal fly ash, and blast furnace slag, which are the main CMs studied and used in alkali-activated materials, so suggest that they are suitable for alkali-activation. With sufficient reactivity of the secondary CMs in these national average cementitious binder mixes, the results indicate that these materials can be classed as low-Ca alkali-activated materials, which

contain alkali aluminosilicate (hydrate) (N-A-S-(H)) gel as a main binding phase, although this requires further research to confirm. We expect some correctives, e.g. clinker, may need to be added to the mixes to adjust their compositions and/or property (compressive strength) development, so the substitution extents (Supplementary Table S1), and hence the resulting GHG emissions reductions from the LCA study, should be treated as upper bounds.

Supplementary Table S3

Oxide compositions for key secondary cementitious materials (CMs).

Secondary CM	SiO_2	CaO	K_2O	P_2O_5	Al_2O_3	MgO	Fe_2O_3	SO_3	Na_2O	TiO_2	CO_2	H_2O	LOI	Mn (ppm)	Sum	Source
Flue gas desulphurization gypsum	2.7	30.3						43.3			2.2	19.5			97.9	[42]
Coal bottom ash[a]	61	1.5	0.2		25.4	1	6.6		0.9						96.6	[42]
Coal fly ash[b]	48	3.2			24.3		15.6	0.4	0.8						92.3	[42]
Granulated blast furnace slag	35	40	0.4		12		1	9	0.3						97.7	[43]
Bauxite residue	9.6	8.6			16.3		40.9		4.5	8.8		10			98.7	[24]
Maize stalk ash	37	13	15		2.4	7.4	1.2	1.3	0.3						77.5	[44]
Maize cob ash	66.4	11.6	4.9		7.5	2.1	4.4	1.1	0.4						98.3	[45]
Rice straw ash	77.2	2.5	12.6	1.0	0.6	2.7	0.5	1.2	1.8	0.04				2790	100	[46]
Rice husk ash	94.5	0.97	2.3	0.5	0.2	0.2	0.2	0.9	0.2	0.02				155	100	[46]
Soybean straw ash	32.6	15.8	21.0	3.7	4.6	8.3	1.5	0.5	0.9				10.7		99.5	[47]
Sugar cane bagasse ash	46.8	4.9	7.0	3.9	14.6	4.6	11.1	3.6	1.6	2.0					100	[46]
Wheat straw ash	50.4	8.2	24.9	3.5	1.5	2.7	0.9	4.2	3.5	0.09				540	100	[46]
Forest residue ash	20.7	47.6	10.2	5.1	3.0	7.2	1.4	2.9	1.6	0.4				13180	100	[46]
End-of-life binder (mortar)[c]	46.6	20.7	0.8	0.1	4.5	1.2	2.9	0.62	0.3	0.2			21.6		99.4	[48]
End-of-life binder (concrete)[c]	46.6	20.7	0.8	0.1	4.5	1.2	2.9	0.62	0.3	0.2			21.6		99.4	[48]

[a] From combustion of bituminous coal.

[b] Class F coal fly ash.

[c] The same oxide composition is used for end-of-life binder in mortar and concrete. This composition is for end-of-life concrete that contains some calcareous and siliceous aggregates in addition to end-of-life cement paste.

Supplementary Table S4

National average cementitious binder mix and cementitious materials (CMs) oxide compositions.

	SiO_2	CaO	Al_2O_3	MgO	Fe_2O_3	SO_3	K_2O	Na_2O	P_2O_5	TiO_2	CO_2	H_2O	LOI (including additional CO_2 and H_2O)[a]	Source
National average cementitious binder mix compositions (mass%)														
Brazil	37.8	17.1	7.9	4.7	8.4	2.5	9.9	1.4	1.7	1.5	0.0	1.5	3.3	Derived here
Canada	41.7	20.7	5.4	2.2	4.1	2.8	5.4	1.0	0.9	0.5	0.1	0.9	12.3	Derived here
China	40.3	20.1	9.5	1.1	6.1	8.0	2.7	0.8	0.2	0.5	0.3	3.1	6.2	Derived here
India	47.1	15.3	7.7	1.6	4.8	7.2	5.4	1.0	0.8	0.3	0.3	2.7	4.9	Derived here
Japan	43.4	22.5	7.4	0.9	3.5	4.9	1.0	0.4	0.1	0.1	0.2	1.3	14.0	Derived here
South Korea	44.0	21.7	6.9	0.9	3.5	4.2	0.8	0.4	0.1	0.1	0.1	1.2	15.8	Derived here
Turkey	43.4	19.9	6.4	1.3	3.6	4.3	3.0	0.6	0.4	0.2	0.1	1.4	14.0	Derived here
United Kingdom	42.3	20.0	5.8	1.3	2.7	4.3	5.7	1.0	0.9	0.1	0.1	0.9	11.0	Derived here
Selected cementitious material oxide compositions (mass%)														
Metakaolin	50.7	2.69	44.6										1.02	[49]
Coal fly ash	42.1	13.6	25.1	1.27	13.2	0.41	0.41	0.81	1.1	1.44				[50]
Blast furnace slag	32.3	42.5	16.3	2.87	2.35								1.91	[49]

[a] LOI is loss on ignition.

S2. Supplementary discussion

S2.1. Life cycle assessment contribution analysis

We performed a contribution analysis to assess the impacts associated with different processing steps on the greenhouse gas (GHG) emissions from cementitious binder production using ReCiPe (2016) 100-year global warming potentials (based on the characterization factors developed by the Intergovernmental Panel on Climate Change) [51]. Our results show that for the GHG emissions from the current (2018) cementitious binder production (Supplementary Fig. S8a), the main contributor is clinker production (93.9% on average) which contributes as high as 95.9% in Brazil and as low as 92.0% in Canada. The second greatest GHG contributor is cement production where blending and mixing of clinker and secondary CMs takes place, contributing about 3.9% on average to the GHG emissions. Cement production here includes impacts attributable to the regional electricity mix, construction of the cement factory, and the production of cement mix constituents (e.g., gypsum, limestone). The third greatest GHG contributor is the transport of cement from cement mill to the batching plant with a share of 1.9% averaged for all countries. The fourth greatest contributor is the transport of secondary CMs to the cement mill which, on average, is responsible for 0.3% of GHG emissions from cradle-to-gate cement production (Supplementary Fig. S8a).

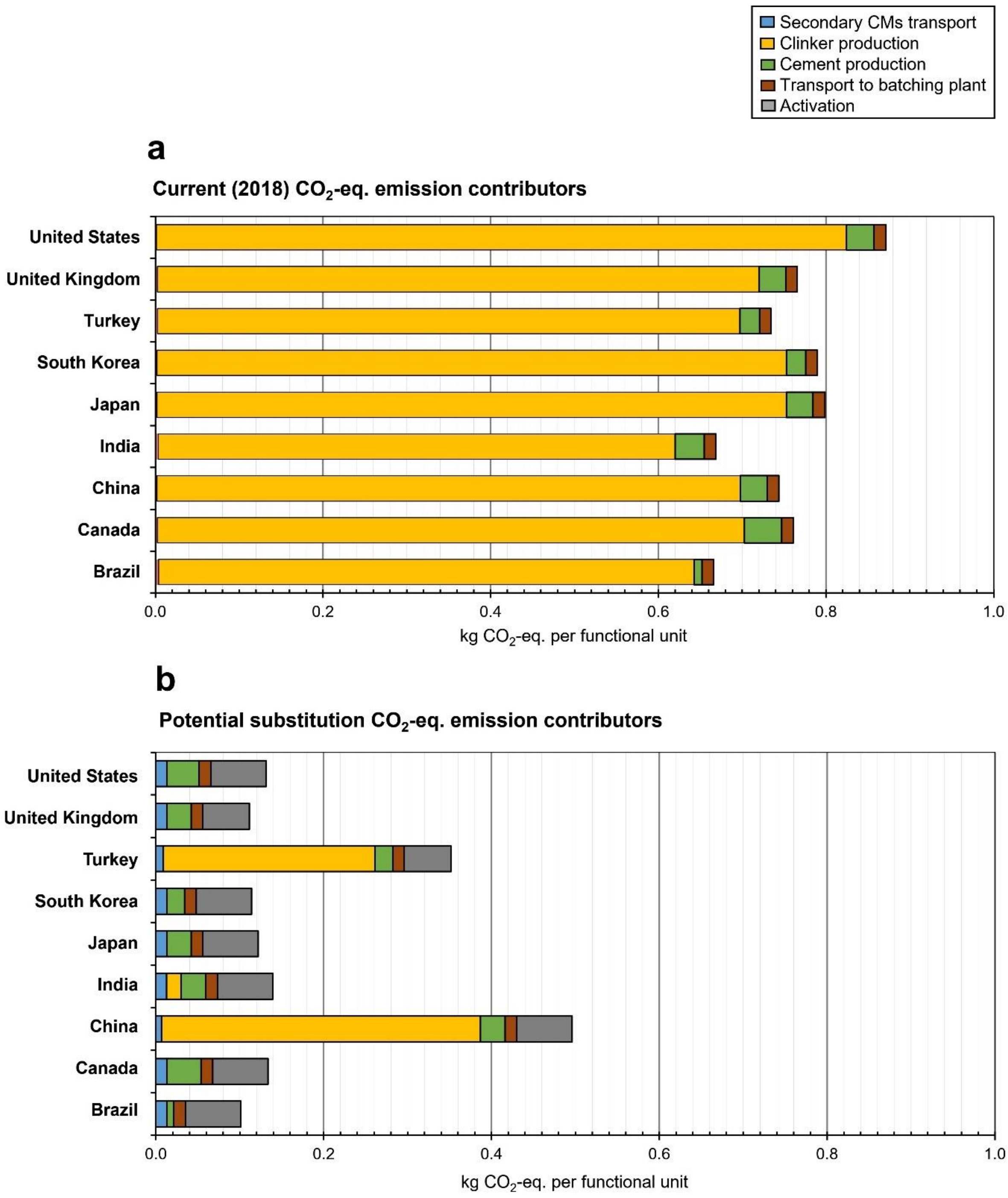

Supplementary Fig. S8. Contribution analysis showing the cradle-to-gate impacts of different processes on GHG emissions (in kg CO_2-eq. per functional unit, i.e., 1.4 kg cementitious binder) from: **a**, current (2018) cement production and **b**, potential cement production with maximal substitution of clinker with secondary CMs.

Our results show that when maximal substitution of clinker for secondary CMs takes place, GHG emissions (Supplementary Fig. S8b) from clinker production are avoided in countries that can fully substitute clinker (i.e., Brazil, Canada, Japan, South Korea, United Kingdom, and the United States). However, for countries able to partially replace clinker (i.e., China, India, and Turkey), clinker production is still a significant contributor to GHG emissions. The activation step is a major contributor to GHG emissions for the high secondary CMs-containing cementitious binders, though its share could be as low as 13.3% in China and as

high as 65.1% in Brazil (with an average of 44.8% for all countries), as shown is Supplementary Fig. S8b. Transport of cements to the concrete batching plant for activation contributes 9.8%, while transport of secondary CMs to the cement mill contributes 8.8% to the cradle-to-gate GHG emissions averaged for all countries (Supplementary Fig. S8b).

Lastly, three important factors should be considered when interpreting these results. First, transport of 150 km was assumed from the site of secondary CM generation to the cement plant. In practice, these distances can be significantly higher, for example for inter-state or international transport. Second, the ecoinvent database has limitations such geographical representativeness and up-to-date process-related data, especially for developing countries, and the associated uncertainty needs to be considered when interpreting our results. Third, our assumption of allocating zero impacts to secondary CMs neglects the generally relatively small impacts attributable to these materials due to their (i) processing from by-products or wastes into secondary CMs and (ii) non-zero costs, the latter being especially relevant for high-demand industrial by-products such as blast furnace slag. Taking the latter factor into account would slightly reduce the decarbonisation potential of cementitious substitution with secondary CMs. We carried out a sensitivity analysis to assess the effect of processing secondary CMs on our results, which we present in the following Section (S2.3).

S2.2. Life cycle assessment sensitivity analysis

We performed a sensitivity analysis whereby all secondary CMs were assumed to undergo an equivalent treatment to that of fly ash, using the inventory data in [52], to indicate the effect that such treatments may have on our LCA results. The key result of this sensitivity analysis shows that, in general, treatment of secondary CMs contributes slightly to the CO_2-eq. emissions of cementitious binder production (Supplementary Fig. S9).

The climate change impact of secondary CM treatment differs for each country depending on its energy inputs (i.e., the electricity and fuel mixes) and the amounts of secondary CMs used. For cementitious binder production in 2018 (Supplementary Fig. S9a), inclusion of these treatment impacts increases CO_2-eq. emissions by between 0.6 g (United States) and 3 g (India) per functional unit (1.4 kg cementitious binder), corresponding to changes of between +0.1 and +0.4%, respectively. For the cementitious binders containing maximal substitution of clinker with secondary CMs, CO_2-eq. emissions from secondary CM treatment are relatively higher per functional unit since they use higher quantities of secondary CMs (Supplementary Fig. S9b). In such cases, we calculate secondary CM treatment to increase CO_2-eq. emissions by between 3 g (Canada) and 11 g (India) per functional unit, corresponding to changes of between +2.5% and +7.7%, respectively. We expect these climate change impacts to vary depending on the secondary CM, although consider fly ash treatment to be a sufficiently reliable proxy for this indicative analysis.

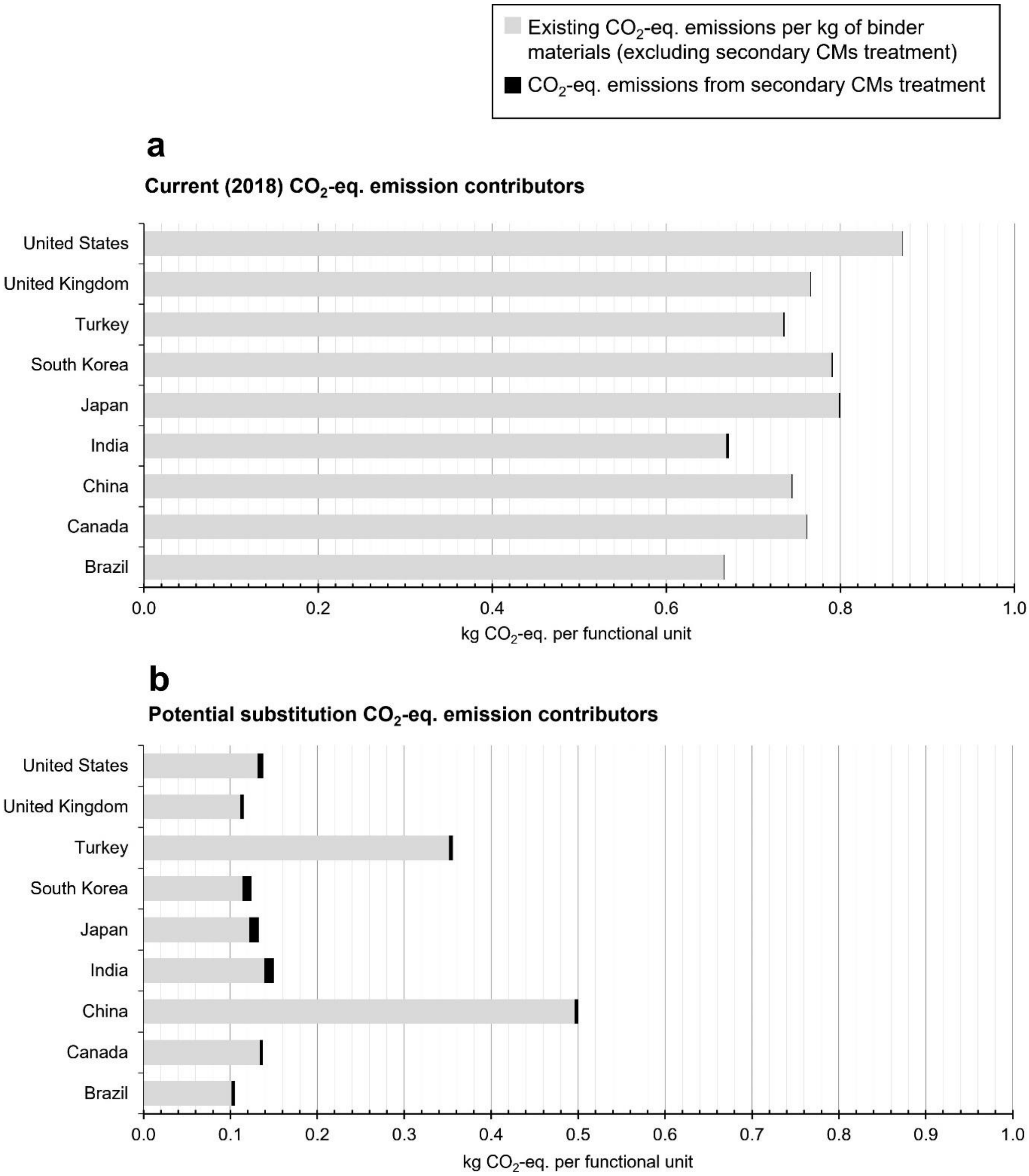

Supplementary Fig. S9. CO_2-eq. emissions per functional unit (1.4 kg cementitious binder) including secondary cementitious material (CM) treatment, for: **a**, current (2018) cement production, and **b**, potential cement production with maximal substitution of clinker with secondary CMs.

S3. Supplementary references

1. Davis, R. E., Kelly, J. W., Davis, H. E. & Carlson, R. W. Properties of cements and concretes containing fly ash. *ACI J. Proc.* **33**, (1937).
2. UK Quality Ash Association. UK quality ash association statistics. http://www.ukqaa.org.uk/information/statistics/ (2020).
3. ACAA. American coal ash association. https://acaa-usa.org/ (2020).
4. BSI. BS EN 197-1:2011: Cement: Part 1: Composition, specifications and conformity

criteria for common cements. (2011).

5. USGS. Mineral commodity summaries. https://www.usgs.gov/centers/nmic/mineral-commodity-summaries (2020).
6. International Energy Agency. Market report series: Coal. https://www.iea.org/reports/coal-2018 (2018).
7. Monticello, D. J. & Finnerty, W. R. Microbial desulfurization of fossil fuels. *Annu. Rev. Microbiol.* **39**, 371–389 (1985).
8. Chandra Srivastava, V. An evaluation of desulfurization technologies for sulfur removal from liquid fuels. *RSC Adv.* **2**, 759–783 (2012).
9. Al Sasi, B. O. & Demirbas, A. Removal of sulfur from sulfur-bearing natural gas to produce clean jet fuel. *Pet. Sci. Technol.* **34**, 1550–1555 (2016).
10. UNEP. International resource panel: Global material flows database. https://www.resourcepanel.org/global-material-flows-database (2020).
11. International Energy Agency. Data and statistics. https://www.iea.org/data-and-statistics (2020).
12. Poullikkas, A. Review of design, operating, and financial considerations in flue gas desulfurization systems. *Energy Technol. Policy* **2**, 92–103 (2015).
13. Ober, J. A., Apodaca, L. E. & Crangle, R. D. Industrial minerals and sustainability: By-products from SO2 mitigation as substitutes for mined mineral commodities. in *Geoscience for the Public Good and Global Development* vol. 520 79–87 (Geological Society of America, 2016).
14. ACAA. Coal combustion products production and use reports. https://acaa-usa.org/publications/production-use-reports/ (2020).
15. USGS. Iron and steel slag statistics and information. https://www.usgs.gov/centers/nmic/iron-and-steel-slag-statistics-and-information?qt-science_support_page_related_con=0#qt-science_support_page_related_con (2021).
16. De Belie, N., Soutsos, M. & Gruyaert, E. *Properties of Fresh and Hardened Concrete Containing Supplementary Cementitious Materials*. vol. 25 (Springer International Publishing, 2018).
17. Pauliuk, S., Milford, R. L., Müller, D. B. & Allwood, J. M. The steel scrap age. *Environ. Sci. Technol.* **47**, 3448–3454 (2013).
18. Wang, X. *et al.* Effect of silica fume particle dispersion and distribution on the performance of cementitious materials: A theoretical analysis of optimal sonication treatment time. *Constr. Build. Mater.* **212**, 549–560 (2019).
19. Diamond, S. & Sahu, S. Densified silica fume: Particle sizes and dispersion in concrete. *Mater. Struct.* **39**, 849–859 (2006).
20. Mari Kirkebøen Næss. Mechanisms and kinetics of liquid silicon oxidation. (Norwegian University of Science and Technology, 2013).
21. Næss, M. K., Tranell, G., Olsen, J. E., Kamfjord, N. E. & Tang, K. Mechanisms and kinetics of liquid silicon oxidation during industrial refining. *Oxid. Met.* **78**, 239–251 (2012).
22. Per Fidjestøl & Magne Dåstøl. *The history of silica fume in concrete-from novelty to key ingredient in high performance concrete*. http://www.ibracon.org.br/eventos/50cbc/plenarias/PER_FIDJESTOL.pdf (2021).
23. European Aluminum. *Environmental profile report - Life-cycle inventory data for aluminium production and transformation processes in Europe*. https://www.european-aluminium.eu/resource-hub/environmental-profile-report-2018/ (2018).
24. Pontikes, Y. & Angelopoulos, G. N. Bauxite residue in cement and cementitious applications: Current status and a possible way forward. *Resour. Conserv. Recycl.* **73**,

53–63 (2013).
25. Food and Agriculture Organization. FAOSTAT. http://www.fao.org/faostat/en/#data/QC (2020).
26. Lal, R. World crop residues production and implications of its use as a biofuel. *Environ. Int.* **31**, 575–584 (2005).
27. Mehta, P. K. Properties of blended cements made from rice husk ash. *J. Am. Concr. Inst.* **74**, 440–442 (1977).
28. Miller, S. A., Cunningham, P. R. & Harvey, J. T. Rice-based ash in concrete: A review of past work and potential environmental sustainability. *Resour. Conserv. Recycl.* **146**, 416–430 (2019).
29. Koopmans, A. & Koppejan, J. Regional consultation on modern applications of biomass energy. in *Proceedings of the Regional Expert Consultation on Modern Applications of Biomass Energy* (1997).
30. Chowdhury, S., Mishra, M. & Suganya, O. The incorporation of wood waste ash as a partial cement replacement material for making structural grade concrete: An overview. *Ain Shams Eng. J.* **6**, 429–437 (2015).
31. Pamenter, S. & Myers, R. J. Decarbonizing the cementitious materials cycle: A whole-systems review of measures to decarbonize the cement supply chain in the UK and European contexts. *J. Ind. Ecol.* **25**, 359–376 (2021).
32. Mineral Products Association. The contribution of recycled and secondary materials to total aggregates supply in Great Britain (2019).
33. Townsend, T. G., Ingwersen, W. W., Niblick, B., Jain, P. & Wally, J. CDDPath: A method for quantifying the loss and recovery of construction and demolition debris in the United States. *Waste Manag.* **84**, 302–309 (2019).
34. Cao, Z., Shen, L., Løvik, A. N., Müller, D. B. & Liu, G. Elaborating the history of our cementing societies: An in-use stock perspective. *Environ. Sci. Technol.* **51**, 11468–11476 (2017).
35. J. Olsson, Miller, S. A. & Alexander, M. Engineering design to mitigate greenhouse gas emissions from cement and concrete (under preparation).
36. Cao, Z. *et al.* The sponge effect and carbon emission mitigation potentials of the global cement cycle. *Nat. Commun.* **11**, 1–9 (2020).
37. United Nations. *World population prospects: The 2017 revision, key findings and advance tables*. https://esa.un.org/unpd/wpp/publications/files/wpp2017_keyfindings.pdf (2017).
38. UNDP. Human development data center. http://hdr.undp.org/en/data (2020).
39. Burhan, S., Srocka, M., Ciroth, A. & Recanati, F. *Ecoinvent version 3.6 in openLCA.* (2019).
40. GCCA. Getting the number right (GNR) project. https://gccassociation.org/gnr/ (2020).
41. Myers, R. J., Bernal, S. A., Gehman, J. D., Van Deventer, J. S. J. & Provis, J. L. The role of al in cross-linking of alkali-activated slag cements. *J. Am. Ceram. Soc.* **98**, 996–1004 (2015).
42. We Energies. *Coal combustion products utilization handbook.* https://www.we-energies.com/environment/pdf/ccp_handbook.pdf (2013).
43. Taylor, H. F. W. *Cement chemistry.* (Thomas Telford Publishing, 1997). doi:10.1680/CC.25929.
44. Aksoɪan, O., Binici, H. & Ortlek, E. Durability of concrete made by partial replacement of fine aggregate by colemanite and barite and cement by ashes of corn stalk, wheat straw and sunflower stalk ashes. *Constr. Build. Mater.* **106**, 253–263 (2016).

45. Adesanya, D. A. & Raheem, A. A. A study of the workability and compressive strength characteristics of corn cob ash blended cement concrete. *Constr. Build. Mater.* **23**, 311–317 (2009).
46. Vassilev, S. V., Baxter, D., Andersen, L. K. & Vassileva, C. G. An overview of the chemical composition of biomass. *Fuel* **89**, 913–933 (2010).
47. Šupić, S., Malešev, M., Radonjanin, V., Bulatović, V. & Milović, T. Reactivity and pozzolanic properties of biomass ashes generated by wheat and soybean straw combustion. *Materials (Basel).* **14**, 1004 (2021).
48. Diliberto, C., Lecomte, A., Mechling, J. M., Izoret, L. & Smith, A. Valorisation of recycled concrete sands in cement raw meal for cement production. *Mater. Struct.* **50**, 1–12 (2017).
49. Bernal, S. A., Rodríguez, E. D., Mejía De Gutiérrez, R., Gordillo, M. & Provis, J. L. Mechanical and thermal characterisation of geopolymers based on silicate-activated metakaolin/slag blends. *J. Mater. Sci.* **46**, 5477–5486 (2011).
50. Walkley, B., Kashani, A., Sani, M. A., Ngo, T. D. & Mendis, P. Examination of alkali-activated material nanostructure during thermal treatment. *J. Mater. Sci.* **53**, 9486–9503 (2018).
51. Stocker, T. F. *et al.* Climate change 2013: The physical science basis. *Working group I contribution to the fifth assessment report of the Intergovernmental Panel on Climate Change* (2014) doi:10.1017/CBO9781107415324.
52. Gursel, A. P. Life-cycle assessment of concrete: Decision-support tool and case study application. (University of California, Berkeley, 2014).
