# Supplementary material for: Cement substitution with secondary materials can reduce annual global CO2 emissions by up to 1.3 gigatons
Source: Nat Commun. 2022 Sep 30;13:5758. doi: 10.1038/s41467-022-33289-7 (PMC9525259; doi:10.1038/s41467-022-33289-7)
Supplement: Supplementary file 3 — Description to Additional Supplementary Information [file 41467_2022_33289_MOESM3_ESM.pdf]

Description of Additional Supplementary Files

Supplementary Data 1 is a spreadsheet that includes the data and results presented here in tabular format.
